# Dietary Polyphenols Decrease Chemokine Release by Human Primary Astrocytes Responding to Pro-Inflammatory Cytokines

**DOI:** 10.3390/pharmaceutics15092294

**Published:** 2023-09-07

**Authors:** Mikolaj Grabarczyk, Dominika Ksiazek-Winiarek, Andrzej Glabinski, Piotr Szpakowski

**Affiliations:** Department of Neurology and Stroke, Medical University of Lodz, ul. Zeromskiego 113, 90-549 Lodz, Polandandrzej.glabinski@umed.lodz.pl (A.G.)

**Keywords:** astrocytes, chemokines, chrysin, dietary polyphenols, inflammation, myricetin

## Abstract

Astrocytes are considered to be the dominant cell fraction of the central nervous system. They play a supportive and protective role towards neurons, and regulate inflammatory processes; they thus make suitable targets for drugs and supplements, such as polyphenolic compounds. However, due to their wide range, knowledge of their anti-inflammatory potential remains relatively incomplete. The aim of this study was therefore to determine whether myricetin and chrysin are able to decrease chemokine release in reactive astrocytes. To assess the antioxidant and anti-inflammatory potential of polyphenols, human primary astrocytes were cultured in the presence of a reactive and neurotoxic astrocyte-inducing cytokine mixture (TNF-α, IL-1a, C1q), either alone or in the presence of myricetin or chrysin. The examined polyphenols were able to modify the secretion of chemokines by human cortical astrocytes, especially CCL5 (chrysin), CCL1 (myricetin) and CCL2 (both), while cell viability was not affected. Surprisingly, the compounds did not demonstrate any antioxidant properties in the astrocyte cultures.

## 1. Introduction

The two main populations of cells within the central nervous system (CNS) are neurons and glial cells. Glial cells outnumber neurons, and divide further into three main subgroups: astrocytes, oligodendrocytes and microglia [1]. Of these, astrocytes are the most abundant, and their main function is to maintain a stable environment for neurons. This includes providing the required energetic supply, secreting neurotrophins crucial for the development of neuronal circuits, and forming glial scars in the event of serious trauma [1,2,3]. They also form part of the tripartite synapse [4]. Their projections may come into contact with pre- and post-synaptic membranes, influencing synapse activity via various secreted factors. Astrocyte cells provide appropriate conditions for synaptic signaling, as they are responsible for the fast reuptake of neurotransmitters used by neurons in synapses. The presence of neurotransmitter-binding and -transporting channels allows astrocytes to clean the extracellular space of numerous neurotransmitters, i.e., serotonin (SERT—serotonine transporter) [5], dopamine (DAT—dopamine transporter) [6], GABA (GATs—gamma-aminonobutyric acid transporters) [7], and glutamate (EAATs—excitatory amino acid transporter) [8,9]. Additionally, it is possible for astrocytes themselves to take part in signaling via a mechanism based on intracellular calcium ion flow [10]. Astrocytes also play an important role in the formation of the blood–brain barrier (BBB). Together with the endothelial cells of vessels within the CNS, and their accompanying pericytes, they regulate the intensity of blood flow and the degree at which individual molecules transmigrate; they also serve as the source of signals involved in initiating the immunological response to hazards that have entered the circulation [11,12,13]. The presence of innate immunity proteins like scavengers, nucleotide oligomerisation domain-like receptors (NLR), Toll-like receptors (TLR) or mannose receptors is important for astrocytes’ signaling [14]. The stimulation of these receptors leads to increased activity of intracellular transmission pathways, such as the nuclear factor kappa-light-chain-enhancer of activated B cells (NF-κB) pathway, and is reflected by heightened cytokine or chemokine secretion. Such stimulation also increases the sensitivity to other immunological mediators [15]. However, different stimulants may induce considerably different changes in astrocyte physiology. One can distinguish two opposite variants, acquired with diverse signals and described as A1 and A2 astrocytes. A1 astrocytes have impaired synaptic and phagocytic functions, also they are highly neurotoxic and pro-inflammatory [16], while A2 astrocytes present a more anti-inflammatory cytokine profile, with increased secretion of beneficial neurotrophins and possible anti-excitotoxic activity [17]. However, this classification is quite simplified, as astrocytes tend to adopt only some of the characteristics of these states, instead of completely polarizing into one of them [18].

Targeting the functional state of astrocytes through future interventions might prove revolutionary in some psychiatric and neurological illnesses. Polyphenols are natural compounds derived from plant tissues with multiple beneficial impacts on numerous organs. The base structure of polyphenols includes at least two phenyl rings connected by different elements and a variable number of hydroxyl substituents. Four main groups can be distinguished depending on the carbonic skeleton construction, namely (1) phenolic acids, (2) flavonoids, (3) stilbenes and (4) lignans, with flavonoids being the most abundant in a typical, human diet [19,20,21]. It has been shown that polyphenols may contribute to a reduction in total serum cholesterol level and counteract low-density lipoprotein (LDL) oxidation [22,23]. They also have a complex protective influence within the CNS depending on both their direct action on neurons and astrocytes, and their indirect actions based on improving blood flow or microbiota composition [24].

Numerous polyphenols exhibit anti-inflammatory and antioxidant potential. They are also known to have a pleiotropic effect on immune mechanisms, manifested as decreased activity of cyclooxygenase (COX) enzymes, the regulation of cytokine and chemokine secretion profiles, the suppression of NF-κB signaling pathway and the up-regulation of suppressor T-cell counts in most types of tissues in human organisms [25,26,27]. This in turn may further contribute to the prevention of DNA damage, the promotion of apoptosis in cancer cells, the induction of remission in autoimmune diseases and a positive influence in age-related cognitive impairment [28,29,30,31]. Some of the compounds that may be worth further examination in terms of neuroprotection include myricetin and chrysin. Myricetin, a polyhydroxyflavonol, takes the form of yellow crystals and can be found in honey, wine, tea and many vegetables and fruits. It has been found to soothe neurological impairment caused by iron and increased acetylcholinesterase (AChE) activity in a mouse model of Alzheimer’s disease [32]. Also, it can prevent oligomerisation of α-synuclein, the main element of Lewy bodies, which are deposits typical for two neurodegenerative diseases: Parkinson’s disease and dementia with Lewy bodies [33]. Additionally, it has been reported to normalise levels of brain-derived neurotrophic factor (BDNF) within the hippocampus of mice exposed to chronic stress [34] and inhibit DNA damage caused by peroxynitrite [35]. Chrysin, on the other hand, is classified as a flavonoid and may be found in honey, passion fruit and some types of mushrooms [36]. It is capable of reducing the levels of inflammation markers in hepatic encephalopathy [37] and alleviating damage and cognitive deficits caused by chronic cerebral hypoperfusion in rat models [38]. There are also reports of its mitigating effect observed in an animal model of Guillain–Barré syndrome (GBS) [39,40]. The data concerning the extensive potential of myricetin and chrysin as neuroprotective and immunomodulatory factors suggest that they are very appealing in terms of their possible usage, as at least supportive treatments, in neurological illnesses. However, these data were acquired mostly with the use of rodent models, and may not entirely reflect the responses of human cells in analogical layouts. Hence, this study examines more specifically how myricetin and chrysin may affect the profile of chemokines secreted by native human astrocytes, either following stimulation with proinflammatory mediators or not, and determines whether these polyphenols are capable of inducing antioxidative mechanisms. Indeed, disturbances in the mechanisms of chemokine release and stimulation of antioxidative processes form the basis of many neurological disorders [41,42,43]. Moreover, as the antioxidant capabilities of polyphenols are quite well established in the available scientific literature, the impact of these compounds on intercellular signaling systems, based on chemokines activity within CNS, remains unknown, because of the very limited data on this topic.

## 2. Materials and Methods

### 2.1. Preparation of Human Astrocyte Cell Cultures

Astrocyte cells were acquired from 3 donors, aged between 18 and 22 weeks, and were provided by ScienCell Research Laboratories Inc. (San Diego, CA, USA). The cells were cultured on 75 cm^2^ culture flasks, covered with poly-L-lysine (ScienCell Research) in astrocyte culture medium (#1801), supplemented with 2% fetal bovine serum (FBS, #0010), antibiotics (penicillin/streptomycin, #0503) and astrocyte growth supplement (AGS, #1852): all supplements were obtained from ScienCell Research Laboratories. Cells were grown at 37 °C in 5% CO_2_ atmosphere with increased humidity. The culture medium was replaced every 72 h; when the cells obtained 70% confluence, the culture medium was changed daily. After reaching 90% confluence, the cells were collected using 0.025% Trypsin/EDTA (ethylenediaminetetraacetic acid) solution in Dulbecco’s PBS, rinsed with PBS/10% FBS and centrifuged (150× *g,* 5 min, 20 °C). Astrocytes were counted in a Bürker chamber and seeded in equal numbers on 48-well (1.3 × 10^5^/well) or 96-well (4.0 × 10^4^/well) culture plates, previously covered with poly-L-lysine. Then, they were left for 24 h to adhere to the growth surface.

### 2.2. Measurement of the Toxicity and Antioxidative Activity of the Polyphenols

Astrocyte cell cultures from 96-well plates, from each donor, were divided into 4 groups incubated in different conditions. The first group was treated with one of the polyphenols, myricetin or chrysin, at concentrations of 0.1 nM, 1 nM, or 5 nM, immediately after seeding the cells. The second group received the same stimulation pattern, but after 24 h, H_2_O_2_ (Merck Millipore, Darmstadt, Germany) was added at a 1 mM concentration. The third and fourth groups were used as controls; these consisted of astrocytes treated with the medium alone or 1 mM hydrogen peroxide. Forty-eight hours after seeding, the number of cells was assessed via the EZMTT assay (Merck Millipore, Darmstadt, Germany). The whole procedure was carried out in accordance with the manufacturer’s instructions. Briefly, after incubation, the culture medium was removed, cells were washed with 200 µL of PBS, and 100 µL of fresh culture medium containing EZMTT reagent (diluted 200-times) was added. Next, the plates were incubated for two hours at 37 °C under 5% CO_2_. After incubation, the absorbance was measured at a wavelength λ = 450 nm. Viability was expressed as the percentage of the absorbance measured for cells cultured in medium alone.

### 2.3. Stimulation of Astrocyte Cultures with Proinflammatory Cytokines and Polyphenols

Twenty-four hours after seeding, the cells from the 48-well plates were divided into 4 groups and incubated under different conditions. The first group was treated with a cocktail of cytokines with proinflammatory activity, described by Liddelow et al. [16] to induce reactive and neurotoxic astrocytes. The mix consisted of 3 cytokines: recombinant human tumor necrosis factor alpha (rhTNF-α) (30 ng/mL, R&D Systems, Ixonia, WI, USA), recombinant human interleukin 1 (rhIL-1a) (3 ng/mL, R&D Systems), and human complement component 1q (hC1q) (400 ng/mL, MyBiosource, San Diego, CA, USA). The production of numerous chemokines by human astrocytes subjected to these pro-inflammatory stimuli has been described elsewhere [44].

The astrocytes of the second group were treated with myricetin or chrysin (both from Merck Millipore, Darmstadt, Germany) at concentrations of 0.1 nM, 1 nM or 5 nM. The third group was initially stimulated with polyphenols (identically as in the previous setup) and after 24 h treated with mixture of proinflammatory cytokines (as in the first setup). The fourth group was cultured only in medium and used as a control. After 6 days of incubation, the astrocyte cultures were examined under the microscope, and their medium was collected, centrifuged (5000× *g*, 10 min, 20 °C), aliquoted and stored at −80 °C for future measurements. These procedures were conducted for astrocytes from three donors.

### 2.4. Measurement of Astrocyte Chemokine Secretion in Response to Proinflamatory Cytokines and Polyphenols

The collected astrocyte medium was thawed and tested for selected chemokine content. Concentrations of CCL1, CXCL1 and CXCL13 chemokines were assessed using the ELISA DuoSet assay (R&D Systems, Mineapolis, MN, USA), and CXCL10, CCL2, CCL5 levels were determined with ELISA kits purchased from Biolegend (San Diego, CA, USA). All the procedures were carried out in accordance with the manufacturer’s instructions. Briefly, the tests were performed on 96-well polystyrene half-area plates, with a microlon high-binding surface (Greiner Bio-one, Kremsmünster, Austria). The wells were covered with capture monoclonal antibody overnight. The plates were then washed 3 times with wash buffer (PBS/0.05% Tween20) and blocked with PBS/1% BSA for 1 h. Following this, the plates were washed, and the samples and standard concentrations were added (50 µL/well). After a 2 h incubation and subsequent washing, monoclonal biotinylated antibodies were added for another 2 h. In the next step, horseradish peroxidase conjugated with avidin (R&D Systems) or streptavidin (Biolegend) was added. After 20 min of incubation and washing, ELISA substrate solution containing 3,3′,5,5′ tetramethylbenzidine was added (Merck Millipore); after 10 min, the color developed, the reaction was stopped with 1 M H_2_SO_4_, and the absorbance was measured (λ = 450 nm).

### 2.5. Statistical Analysis

Statistical analysis was performed with Statistica 13.1 software (TIBCO Software Inc., Houston, TX, USA). The normality of the distribution was assessed with the Shapiro–Wilk test. Variables with a non-normal distribution were tested using the non-parametric Kruskal–Wallis test followed by the Mann–Whitney U-test. Statistical significance was assumed for *p* < 0.05.

## 3. Results

### 3.1. Neither Myricetin nor Chrysin Affect Human Astrocyte Viability or Proliferation Rate

**Figure 1:**
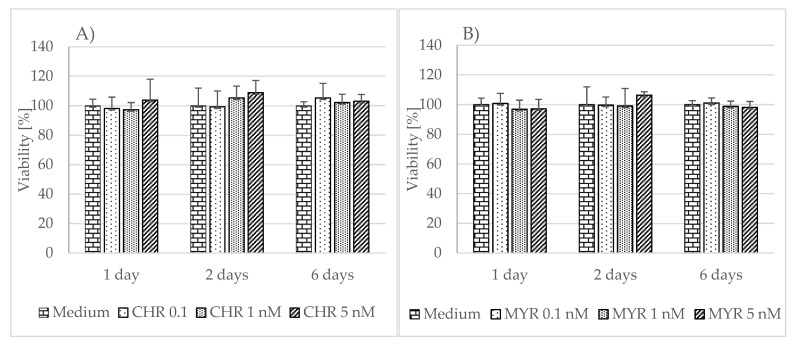
The effect of chrysin (CHR) (**A**) and myricetin (MYR) (**B**) on human astrocyte viability. The results were obtained from 4 separate experiments performed for each of the 3 donors. Cells were cultured on 96-well plates for 24, 48 h or 6 days with each polyphenol at a 0.1 nM, 1 nM, and 5 nM concentration and in non-stimulatory conditions (culture medium). Data are shown as mean viability ± SD. Normality of the distribution was checked with the Shapiro–Wilk test. For comparisons between groups, the Mann–Whitney U-test or Student’s *t*-test was used; differences were considered significant for *p* values < 0.05.

To examine myricetin’s and chrysin’s effect on cells viability and proliferation rate, we used the MTT assay. Neither polyphenolic compound was found to have any significant effect on cell viability or proliferation rate, compared to unstimulated astrocytes at the tested concentrations (Figure 1).

### 3.2. Neither Myricetin nor Chrysin Provide Significant Protection against Oxidative Stress

**Figure 2:**
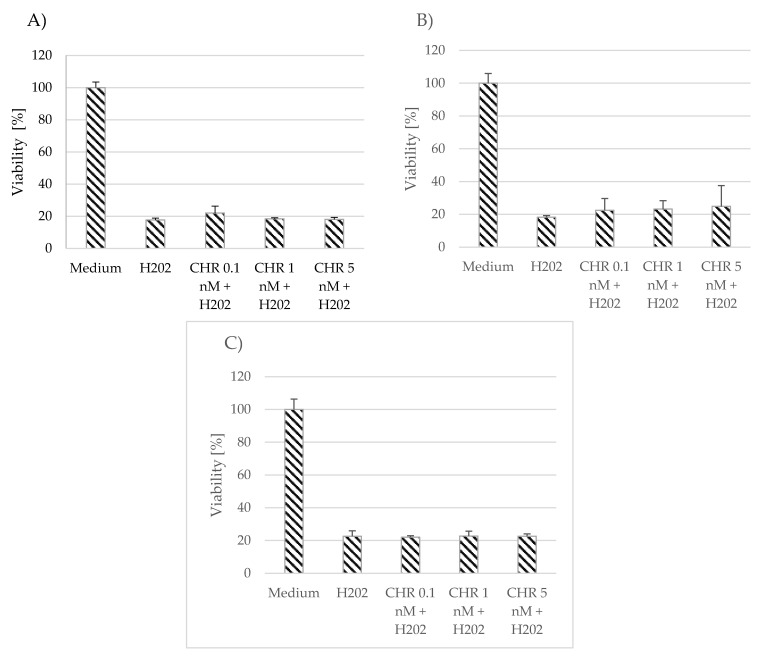
Effect of chrysin (CHR) on cell viability in conditions of oxidative stress. The data were acquired from 4 separate experiments performed for each of the 3 donors: D1 (**A**), D2 (**B**), D3 (**C**). Cells were cultured on 96-well plates for 48 h in non-stimulatory conditions (culture medium), with 1 mM H_2_O_2_, and with 0.1, 1, or 5 nM chrysin (CHR) with the subsequent addition of 1 mM H_2_O_2_. Results are shown as mean viability ± SD. Normality of the distribution was checked with the Shapiro–Wilk test. For comparisons between groups, the Mann–Whitney U-test was used; differences were considered significant for *p* values < 0.05.

**Figure 3:**
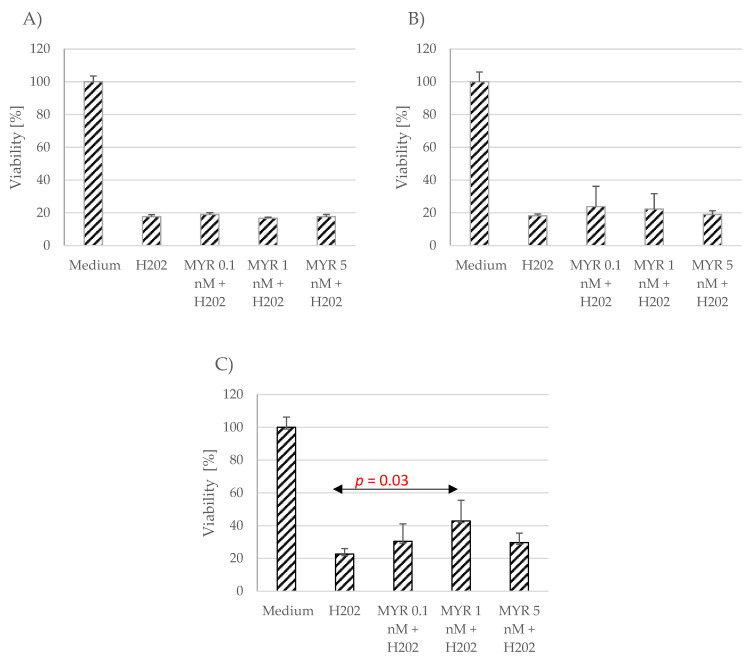
Effect of myricetin (MYR) on cell viability in conditions of oxidative stress. The data were acquired from four separate experiments performed for each of the 3 donors: D1 (**A**), D2 (**B**), D3 (**C**). Cells were cultured on 96-well plates for 48 h in non-stimulatory conditions (culture medium), with 1 mM H_2_O_2_, and with 0.1, 1 nM or 5 nM myricetin (MYR) with the subsequent addition of 1 mM H_2_O_2_. Results are shown as mean viability ± SD. Normality of the distribution was checked with the Shapiro–Wilk test. For comparisons between groups, the Mann–Whitney U test was used; differences were considered significant for *p* values < 0.05.

The effect of myricetin and chrysin on cell viability in conditions of oxidative stress was evaluated using the MTT assay. Neither polyphenolic compound, at the applied concentrations (0.1 nM, 1 nM, 5 nM)), showed any protection against oxidative stress caused by hydrogen peroxide (1 mM), or the effect was minimally visible and nonsignificant compared to unstimulated astrocytes (Figure 2 and Figure 3). However, the applied polyphenol appeared to alleviate the effect induced by hydrogen peroxide in the myricetin-stimulated cells acquired from donor number 3 (*p* = 0.03) (Figure 3C).

### 3.3. Astrocytes Produce Small Amounts of Chemokines in Non-Inflammatory Conditions, Which Can Be Altered Using Myricetin and Chrysin

**Figure 4:**
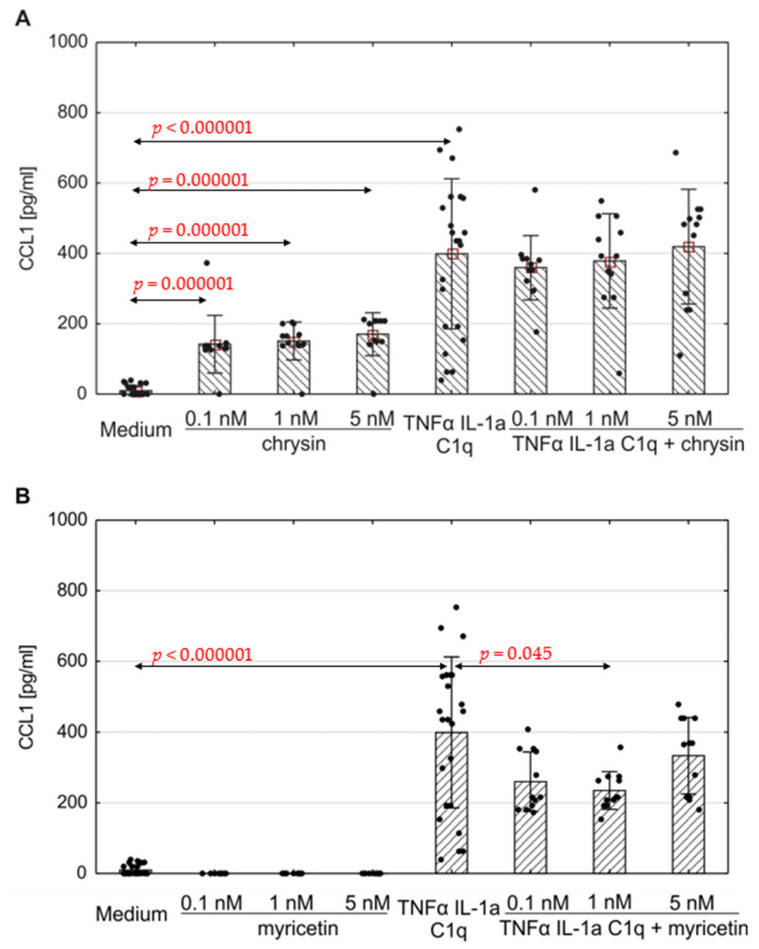
Production of CCL1 by human astrocytes in response to chrysin (**A**) or myricetin (**B**). The results were acquired from at least 4 separate experiments performed for the 3 donors. Cells were cultured on 48-well plates for 6 days in proinflammatory conditions (TNF-α/IL-1a/C1q, 24 results), with chrysin (**A**) or myricetin (**B**) stimulation (respectively, 0.1 nM, 1 nM, 5 nM; 12 results for every point), or under proinflammatory conditions with the addition of chrysin (CHR + TNF-α/IL-1a/C1q, 12 results) or myricetin (MYR + TNF-α/IL-1a/C1q, 12 results) or the culture medium alone (24 results). Data are shown as mean chemokine concentration ± SD. The normality of the distribution was checked with the Shapiro–Wilk test. The groups were compared using the Mann–Whitney U-test; significant differences were assumed for *p* < 0.05. The black dots show the distribution of results within groups.

**Figure 5:**
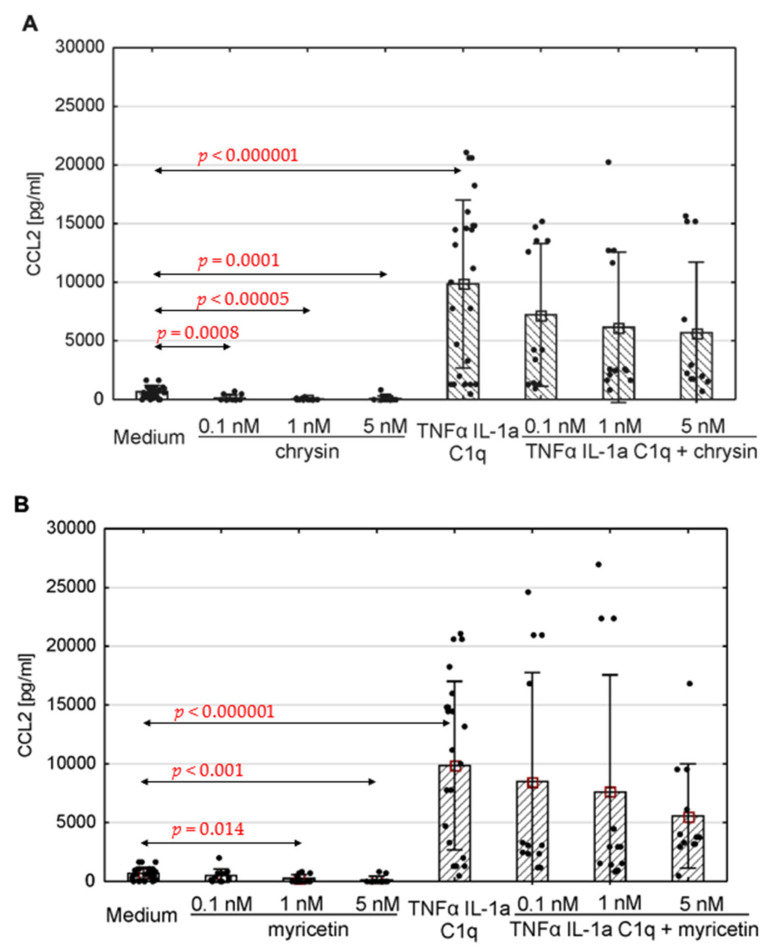
Production of CCL2 by human astrocytes in response to chrysin (**A**) or myricetin (**B**). The results were acquired from at least 4 separate experiments performed for the 3 donors. Cells were cultured on 48-well plates for 6 days in proinflammatory conditions (TNF-α/IL-1a/C1q, 24 results), with chrysin (**A**) or myricetin (**B**) stimulation (respectively, 0.1 nM, 1 nM, 5 nM; 12 results for every point), or under proinflammatory conditions with the addition of chrysin (CHR + TNF-α/IL-1a/C1q, 12 results) or myricetin (MYR + TNF-α/IL-1a/C1q, 12 results) or the culture medium alone (24 results). Data are shown as mean chemokine concentration ± SD. Normality of the distribution was checked with the Shapiro–Wilk test. For comparisons between groups, the Mann–Whitney U-test was used, and differences were considered significant for *p* < 0.05. Black dots show the distribution of results within groups.

**Figure 6:**
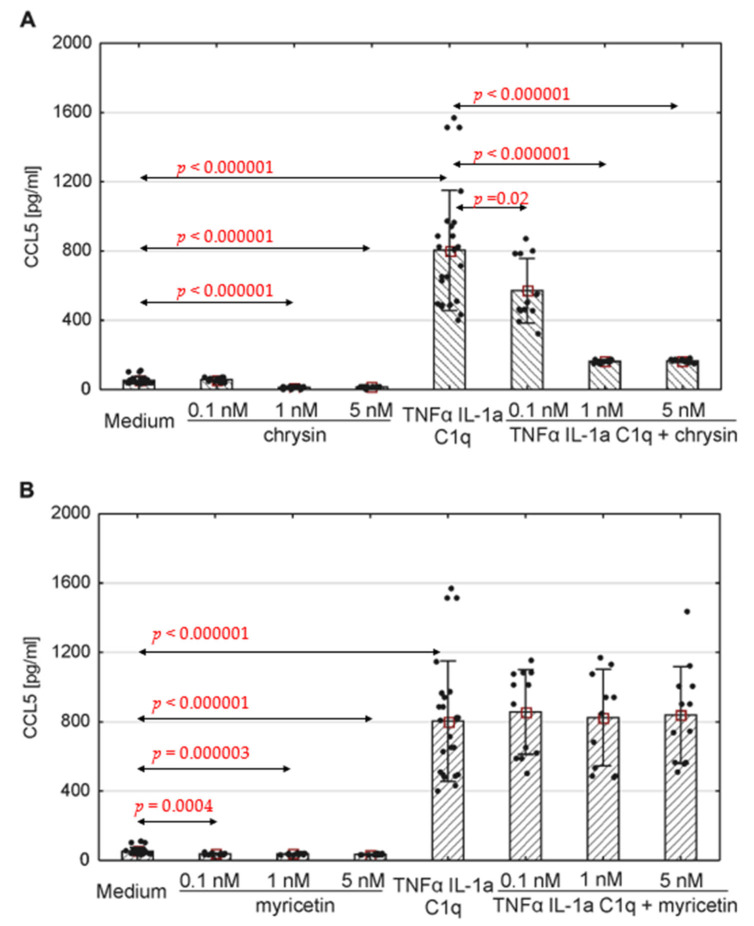
Production of CCL5 by human astrocytes in response to chrysin (**A**) or myricetin (**B**). The results were acquired from at least 4 separate experiments performed for the 3 donors. Cells were cultured on 48-well plates for 6 days in proinflammatory conditions (TNF-α/IL-1a/C1q, 24 results), with chrysin (**A**) or myricetin (**B**) stimulation (respectively, 0.1 nM, 1 nM, 5 nM; 12 results for every point), or under proinflammatory conditions with the addition of chrysin (CHR + TNF-α/IL-1a/C1q, 12 results) or myricetin (MYR + TNF-α/IL-1a/C1q, 12 results) or the culture medium alone (24 results). Data are shown as mean chemokine concentration ± SD. The normality of the distribution was checked with the Shapiro–Wilk test. For comparisons between groups, the Mann–Whitney U-test was used, and differences were considered significant for *p* < 0.05. Black dots show the distribution of results within groups.

The production of selected chemokines was measured to determine the secretory activity of native astrocytes. Spontaneous CCL1, CCL2, CCL5 and CXCL10 release was observed (Figure 4, Figure 5 and Figure 6; Supplementary Figure S2). The addition of polyphenolic compounds modified the release of a few secreted chemokines. Both chrysin and myricetin decreased the basic production of CCL2—chrysin at all examined concentrations (Figure 5A; *p* = 0.0008, *p* = 0.00005, *p* = 0.0001, respectively), and myricetin at 1 nM and 5 nM (Figure 5B; *p* = 0.014, *p* = 0.001, respectively). Also, the basic production of CCL5 was decreased by chrysin (1 nM and 5 nM, Figure 6A; *p* < 0.000001) and myricetin (*p* = 0.0004, *p* < 0.000003, *p* < 0.000001, respectively, for 0.1 nM, 1 nM and 5 nM). Interestingly, chrysin significantly increased the release of CCL1 in all examined doses (*p* < 0.000001) (Figure 4A).

### 3.4. Proinflammatory Stimulation Results in Chemokine Release in Human Astrocytes, Which Can Be Altered by Myricetin and Chrysin

The concentration of the selected chemokines was measured to determine the secretory response of the astrocytes to various pro-inflammatory stimuli. TNF-α/IL-1a/C1q stimulation resulted in a significant increase in all examined chemokines, namely CCL1, CCL2, CCL5, CXCL1, CXCL10, and CXCL13, compared to unstimulated cells (Figure 4, Figure 5 and Figure 6, Supplementary Figures S1–S4); however, the levels varied between donors. Myricetin (1 nM) was able to mitigate the increase in CCL1 driven by pro-inflammatory cytokines (*p* = 0.045) (Figure 4B). The release of other chemokines was more complex due to the high variability between donors, as seen for CXCL1 (Supplementary Figure S2) and CXCL13 (Supplementary Figure S4A). Chrysin, in turn, was effective at decreasing the production of CCL5 (*p* = 0.02 for 0.1 nM, *p* <0.000001 for both 1 nM and 5 nM) in all donors (Figure 6A). It also decreased the release of CCL2; however, the difference was not significant (Figure 5). Surprisingly, chrysin combined with proinflammatory stimuli increased the release of CCL1 depending on the donor (Supplementary Figure S1F), along with CXCL1 (Supplementary Figure S2F) and CXCL10 (Supplementary Figure S3B).

## 4. Discussion

Polyphenols are the most abundant group of phytochemicals and possess a wide variety of potential health benefits. They possess a highly anti-inflammatory and antioxidant activity [45]. They have also been found to demonstrate cardioprotective, anti-ageing and anti-cancer effects. Numerous human and animal studies indicate that dietary polyphenols have beneficial effects on brain function, resulting in improved CREB/BDNF pathway-dependent cognition, increased blood flow, better memory function and language skills, and a protective role against the development of Alzheimer’s or Parkinson’s disease [46,47,48,49,50].

Consumption of specific polyphenols may be a protective factor in terms of age-related brain atrophy [51]. Animal studies suggest that polyphenol intake may be associated with reduced accumulation of amyloid beta (Aβ) plaques and tau protein phosphorylation [52]. Other in vivo studies on rats indicate that polyphenols demonstrate neurorestorative capability after cerebral ischemic injury [53]. It is enormously important to understand the effect of polyphenols on both neuronal and glial cells.

Myricetin is a flavonol present in products of plant origin, such as fruits, vegetables and seeds (tomatoes, oranges) [54]. Chrysin is a plant-derived flavonoid present in plant tissues [36]. Myricetin consumption was proven to have a beneficial impact on the human brain after stroke, and is believed to support CNS regeneration [55]. As no information exists in the literature about the impact of myricetin and chrysin on astrocytes, the aim of the present study was to determine their potential as regulatory molecules in proinflammatory conditions.

This study first assessed the effect of preincubation with both compounds in normal and proinflammatory conditions on the proliferation and viability of astrocytes. Neither myricetin nor chrysin at the studied concentrations seemed to significantly affect the astrocyte proliferation rate, nor present a cytotoxic effect. This is not surprising, as a similar phenomenon has been already observed at higher concentrations for micromolar doses [51,56,57,58].

Polyphenolic compounds are known to have antioxidative properties, which are expressed through direct interactions with prooxidative agents and via the modulation of cellular enzymatic routes engaged in oxidoreductive processes [53,59,60,61]. In our experimental conditions, myricetin and chrysin were not able to significantly protect human astrocytes against oxidative stress induced by H_2_O_2_ treatment, as no significant changes in cell viability were observed; however, one exception was noted, where myricetin induced a small but significant rise in cell viability. While previous studies have found the investigated polyphenols to have strong, direct antioxidative potential in systems composed of only polyphenol and oxidative compounds, these studies were performed using micromolar doses of myricetin or chrysin [62,63,64,65,66]. Similarly, in vitro studies of hamster fibroblasts incubated with micromolar concentrations of myricetin reported increased cell viability and noted the induction of various antioxidant enzymes, including glutathione peroxidase, catalase, superoxide dismutase in conditions of oxidative stress [54,67]. Chrysin also effectively enhanced superoxide dismutase, glutathione peroxidase and glutathione reductase activity in a rat hyperoxia lung injury model [68]. Our experimental setting was based on nanomolar concentrations of polyphenols, as they are more relevant to those detectable within CNS, although data concerning this topic are limited and rarely discussed [60,69].

Apart from antioxidative features, polyphenols have a well-documented anti-inflammatory potential. In relation to autoimmune, infectious and cancerous disorders of CNS, the anti-inflammatory mode of action of polyphenols seems promising. Reactive astrocytes are present within the CNS in numerous neurological diseases, where they contribute to illness development. Such activity can be induced by the presence of active microglia, which promote astrocytes’ neurotoxic and pro-inflammatory phenotype with Il-1α, TNF and C1q. Those cytokines are considered crucial for A1 astrocytes’ polarisation, and thus antagonizing their effect may lead to the mitigation of inflammatory response [16]. The reactive phenotype is related to an alteration of the secretory profile. One large group of signalling particles released by astrocytes are chemokines, whose levels vary according to current astrocyte activity [68,70,71,72]. Most chemokines are considered as markers of astrocytes’ proinflammatory state, and their release may be altered by numerous compounds [73,74]. Polyphenols seem to fall into this category, although few data exist concerning the effect they have on chemokine release in astrocyte cell cultures and animal models. Curcumin and resveratrol has been found to decrease cytokine and chemokine production (mostly CCL2) [75,76,77,78], and to reduce CXCL1 and CXCL2 production in human prostate cancer [61], as well as CCL2, CCL5 and CXCL10 production in murine myocarditis, endometriotic stromal cell and human keratinocyte models, respectively [65,66,79]. However, no data are available regarding the impact of myricetin or chrysin on chemokine expression in astrocytes.

One of the chemokines secreted by astrocytes is CCL1, which induces the migration of cells expressing the CCR8 receptor. It is one of the crucial mechanisms playing a role in inducing Th2 lymphocyte-mediated inflammation [52,80]. CCR8 is also expressed on macrophages, monocytes and epithelial cells [53,81], as well as the most abundant cells in the CNS, viz. neurons, astrocytes and microglia [61,82]. Additionally, CCL1 demonstrates anti-apoptotic activity, mediated through its subordinate enzymatic pathways [79,83]. CCL1 acts on microglia, increasing their motility, proliferation rate and phagocytic capabilities [61,82]. Astrocytes secrete CCL1 after exposure to IL-1α, TNF-α and complement C1q [44,45]. Our present findings indicate that human astrocytes incubated with myricetin released lower amounts of CCL1 after being treated with a proinflammatory cocktail; however, chrysin seems able to increase CCL1 release, similarly to the mentioned cytokines.

Both an increase and decrease in CCL1 secretion could prove beneficial in therapeutic contexts, depending on the treated illness. Many studies reported increased activity of CCL1 in research models and patients suffering from neurological disorders. In a mouse model of multiple sclerosis, high levels of CCL1 in the cerebrospinal fluid (CSF) are associated with increased B-cell and T-cell infiltration of CNS [66,84]. In such cases, interventions focused on downregulating CCL1 release could result in ameliorating the damage caused by transmigrating lymphocytes. Furthermore, this chemokine also induces FoxP3+ regulatory T (Treg) cell migration into the brain, where they take part in neurological recovery after stroke, mostly by reducing astrogliosis [65,85]. Thus, elevating the CCL1 level during medical care could result in better outcomes in patients.

Another tested chemokine produced by human astrocytes was CXCL1. It serves as a chemotactic factor for neutrophils, achieving its biological effect through the CXCR2 receptor [54,86]. It is also capable of stimulating neutrophil release from bone marrow [56,87]. Pro-inflammatory cytokines induce CXCL1 production and release in human astrocytes [57,88]. In our study, proinflammatory cytokines, but not polyphenols, significantly increased CXCL1 level in supernatants from the culture medium of all examined astrocyte donors. Myricetin was able to decrease CXCL1 release after proinflammatory stimulation only in astrocytes taken from donor 1. Interestingly, cells from the same donor produced higher amounts of CXCL1 in response to chrysin. Our findings suggest that the examined polyphenols are not effective in altering CXCL1 secretion; however, they may vary in their mode of action on the CXCL1 expression pathway. A better understanding of the factors capable of reducing CXCL1 expression may prove beneficial in many neurological disorders, as higher levels of this chemokine can be detected in CSF from patients experiencing neuromyelitis optica and stroke [59,89]. In cases of stroke, the levels of CXCL1 also positively correlate with area of ischemic tissue [81,90]. In a murine model of viral encephalitis, blocking CXCL1-dependent signalling leads to reduced BBB permeability, neutrophil penetration and mortality [82,91].

CXCL10 is involved in the recruitment of CD4+, CD8+ and FoxP3+ regulatory T cells. It promotes greater chemotaxis of these immune cells and their polarisation into effector T cells [83,84,92,93]. In the present study, a mixture of TNF-α, IL-1a and C1q induced CXCL10 production by human astrocytes, but, as in the case of CXCL1, production was not significantly affected by the examined polyphenols. However, the cells of donor number 1 demonstrated a stronger reaction to the pro-inflammatory cytokine cocktail following pre-treatment with chrysin. Finding molecules capable of significantly reducing CXCL10 release could improve current therapeutic options for many neurological patients, as many studies highlight its important role in disorders of inflammatory and degenerative origin. Experiments utilizing a mouse cuprizone model have reported that CXCL10 is able to induce a proinflammatory phenotype in microglia [85,94]. Additionally, it has been observed that astrocyte-produced CXCL10 regulates microglial migration toward an injury site, through a CXCR3-mediated mechanism [45]. Moreover, raised serum CXCL10 levels can be associated with a worse outcome after experiencing intracerebral haemorrhage [86,95], and an elevated CXCL10 level in the CSF has been acknowledged as a biomarker of active multiple sclerosis [87,88,96,97]. Conditional ablation of astrocyte-derived CXCL10 results in reduced accumulation of leukocytes in perivascular spaces [37]. This chemokine has been also reported to cause the apoptosis of neurons via mechanisms based on altering intracellular calcium ion flow [91,98].

CXCL13 was the first chemokine found to have a selective chemotactic effect on B cells [92,99]. This chemokine recognises the CXCR5 receptor, and this interaction plays a key role in the regulation of cell migration within secondary lymphoid organs [93,100]. It is also postulated that it serves as one of the crucial factors for the formation of tertiary lymphoid organs, i.e., structures taking the form of immune cell clusters, associated with chronic inflammation [94,101]. Within the CNS, CXCL13 takes part in organizing ectopic lymphoid structures, characterised by increased B cell activity [95,102]. In the present study, a mixture of TNF-α, IL-1a, and C1q was found to have the potential to promote CXCL13 secretion by human astrocytes. Previous addition of myricetin or chrysin was not able to significantly affect pro-inflammatory cytokine-induced CXCL13 release by astrocytes; however, interestingly, the astrocytes from donor 1 preincubated with myricetin demonstrated significantly reduced CXCL13 production under pro-inflammatory conditions, as well as greater CXCL13 production compared with other donors. This might suggest that myricetin is capable of reducing CXCL13 release at generally higher production levels, which seems favourable for maintaining homeostasis.

Discovering methods or molecules able to decrease CXCL13 secretion could expand therapeutic options in demyelinating diseases, as tissue samples from patients with MS and its murine model show increased CXCL13 synthesis in glial cells [97,103,104]. Its levels are also raised in the CSF of MS patients during relapse [96,97,105,106,107,108]. A similar phenomenon occurs in neuromyelitis optica, viral encephalitis and Lyme neuroborreliosis [98,99,109,110,111,112]. In a mouse model of amyotrophic lateral sclerosis, CXCL13/CXCR5 signalling was strongly up-regulated among motor neurons, but silencing the axis resulted in increased motor neuron loss [100,113]. CXCL13 also seems to play an important role in the infiltration of T follicular helper cells into the ischemic tissue after stroke [101,103].

CCL2 is a chemokine primarily secreted by epithelial and endothelial cells, macrophages, fibroblasts, microglia and astrocytes [102,114]. Its most important function is monocyte chemotaxis, but it also takes part in the migration of memory T cells and natural killer cells [105,106,115,116]. In our study, human astrocytes released CCL2 in response to TNF-α, IL-1a, and C1q stimulation. Chrysin and myricetin were found to reduce the basal levels of this chemokine in most analysed donors. Additionally, in cells obtained from donor number 2, incubation with myricetin or chrysin led to lower CCL2 release in response to proinflammatory cytokine treatment. Strategies aimed at reducing CCL2 level could support the treatment of neurodegenerative and demyelinating disorders, as a number of studies indicate that these levels are elevated in the body fluids and tissues of affected patients. In chronic traumatic encephalopathy, this CCL2 activity rises with the illness severity in affected regions of brain tissue. There has also been observed a correlation between tau pathology intensity and CCL2 levels in this disease [107,117]. In a rodent model of experimental autoimmune encephalomyelitis, deletion of the CCL2 gene resulted in a decrease in macrophage and T-cell infiltration, thus ameliorating disease severity in its late stages. This suggests that this chemokine plays an important role in immune cell transmigration into the CNS and the activation of glial cells [111,118]. It has also been found that deletion of the CCL2 gene increases the expression of two anti-inflammatory enzymes, viz. 15-lipoxygenase and 5-lipoxygenase, in the presence of inflammatory stimuli, leading to a reduced inflammatory response in the brain cortex [119,120]. In a rodent model of Alzheimer’s disease, such deletion led to reduced neuronal degeneration and amyloid beta plaque accumulation [113,121]. This might suggest that interventions able to decrease CCL2 release could yield a similar, potentially therapeutic effect.

CCL5 mostly fulfils its biological activity via interaction with the CCR5 receptor. It acts as a chemoattractant mainly for T cells, but it also has an impact on dendritic cells, eosinophils, basophils, NK cells and mastocytes. Its main sources are T cells, macrophages, platelets, fibroblasts, epithelial cells, and numerous tumors [114,115,116,122,123,124]. Our present findings indicate spontaneous secretion of CCL5 by human astrocytes from all donors. Those levels were significantly increased after the cells were treated with pro-inflammatory cytokines (TNF-α, IL-1a, and C1q). Chrysin proved successful in reducing both basic and inflammatory-induced CCL5 release in cells acquired from all donors. Myricetin was less effective as the reduction in both sets of conditions occurred only among the cells from donor number 2, whereas spontaneous CCL5 release was inhibited in astrocytes from donor 1.

CCL5 secretion by astrocytes may induce expression of other cytokines and chemokines, including CCL2, TNF-α and also CCL5 itself [125]. Recent research suggests that blockage of the CCL5/CCR5 axis may be a promising target for recovery after ischemic stroke. Rodent models of such intervention have provided data suggesting that it may restrain tissue death within the brain, partially neutralise inflammatory responses, and increase the amounts of molecules engaged in promoting neural plasticity [117,118,126,127,128,129]. A positive effect has also been observed in a mouse model of epilepsy, where antagonizing the CCL5 activity resulted in lower astrocyte and microglia activation and reduced seizure severity [119,130].

Other potential targets for CCL5 release reduction include demyelinating and infectious diseases, as studies assessing the CSF from people with MS have revealed increased CCL5 levels among patients with an active form of disease [121,131,132,133]. Higher CCL5 amounts are also present in the CSF of HIV-infected patients who developed HIV-associated dementia and patients diagnosed with bacterial meningitis [122,123,134,135].

The potential of myricetin to alleviate inflammation has been studied in numerous injury and inflammatory models [62,136]. One of the signalling pathways believed to be inhibited by myricetin is the receptor activator of nuclear factor kappa-Β ligand/receptor activator of nuclear factor kappa-Β (RANKL/RANK) axis, mostly engaged in the bone remodelling processes [63,137]; However, it is also active within the CNS, where it is responsible for the febrile and inflammatory response of glial and local immune cells [64,138]. It is suggested that this effect is mainly achieved via the stimulation of NF-κB, a typical proinflammatory transcription factor activated through RANKL/RANK axis, believed to be responsible for inducing the expression of several cytokines and chemokines and the promotion of an inflammatory phenotype in various cell types [67,139,140,141]. Numerous studies indicate that myricetin is able to inhibit the NF-κB and MAPK cascades, which may explain its multilevel anti-inflammatory potential. Such inhibition is achieved mainly by reducing the phosphorylation of proteins crucial for those pathways, thus potentially alleviating the TLR-, IL-1- or TNF-α-mediated inflammatory response [142,143,144,145,146,147].

Chrysin also demonstrates broad anti-inflammatory capabilities [120,147,148,149]. It is able to suppress the NF-κB signalling pathway, mainly by decreasing protein phosphorylation and preventing p65 subunit transmission into the nucleus [150,151]. Murine models indicate that, similarly to myricetin, chrysin is able to inhibit the RANKL-promoted NF-κB and MAPK signalling pathways [152,153]. Suppression of the NF-κB and MAPK pathways within astrocytes in neurotoxic and inflammatory conditions is accompanied by decreased CCL2, CCL5, CCL7, and CXCL10 release [145,146,152,154,155,156]. Thus, regulation of these pathways by studied polyphenols might be the main reason for decreased chemokine production observed among cytokine-stimulated astrocytes in our study. However, to prove this hypothesis, more detailed studies are needed.

## 5. Conclusions

Myricetin and chrysin do not appear to significantly affect human astrocyte proliferation or viability in the examined doses, nor do they display any protective effect on H_2_O_2_-induced oxidative stress. However, the investigated polyphenols were able to reduce chemokine secretion under strong pro-inflammatory conditions. Data concerning the influence of polyphenols on chemokine secretion in human astrocytes are very limited, with this being the first such study to assess the effect of myricetin and chrysin on CCL1, CCL2, and CCL5 secretion in reactive astrocytes. Chrysin seems a promising factor for inhibiting CCL5 release by human reactive astrocytes, whereas myricetin lowered CCL1 release. This raises the possibility that these polyphenols may be used as components in therapeutic approaches to treat neuroinflammatory diseases, alone or as drugs derived in liposomes.

## Data Availability

Research data are deposited by the authors.

## Supplementary Materials

The following supporting information can be downloaded at: https://www.mdpi.com/article/10.3390/pharmaceutics15092294/s1, Supplementary Figure S1. Expression of CCL1 in human astrocytes of respective donors: D1 (A,B), D2 (C,D), D3 (E,F). Results were acquired from 4 separate experiments performed for 3 donors. Data shown as mean chemokine concentration ± SD. Cells were cultured on 48-well plates for 6 days in proinflammatory conditions (TNF-α/IL-1a/C1q), myricetin stimulation (MYR), chrysin stimulation (CHR), proinflammatory conditions with addition of myricetin (MYR + TNF-α/IL-1a/C1q), proinflammatory conditions with addition of chrysin (CHR + TNF-α/IL-1a/C1q) and in non-stimulatory conditions (culture medium). Normality of the distribution was checked with Shapiro–Wilk test. For comparisons between groups, Mann–Whitney U test was used and differences were considered significant for *p* values < 0.05. Supplementary Figure S2. Production of CXCL1 in human astrocytes of respective donors: D1 (A,B), D2 (C,D), D3 (E,F). Results were acquired from 4 separate experiments performed for 3 donors. Data shown as mean chemokine concentration ± SD. Cells were cultured on 48-well plates for 6 days in proinflammatory conditions (TNF-α/IL-1a/C1q), myricetin stimulation (MYR), chrysin stimulation (CHR), proinflammatory conditions with addition of myricetin (MYR + TNF-α/IL-1a/C1q), proinflammatory conditions with addition of chrysin (CHR + TNF-α/IL-1a/C1q) and in non-stimulatory conditions (culture medium). Normality of the distribution was checked with Shapiro–Wilk test. For comparisons between groups, Mann–Whitney U test was used and differences were considered significant for *p* values < 0.05. Supplementary Figure S3. Production of CXCL10 in human astrocytes of respective donors: D1 (A,B), D2 (C,D), D3 (E,F). Results were acquired from 4 separate experiments performed for 3 donors. Data shown as mean chemokine concentration ± SD. Cells were cultured on 48-well plates for 6 days in proinflammatory conditions (TNF-α/IL-1a/C1q), myricetin stimulation (MYR), chrysin stimulation (CHR), proinflammatory conditions with addition of myricetin (MYR + TNF-α/IL-1a/C1q), proinflammatory conditions with addition of chrysin (CHR + TNF-α/IL-1a/C1q) and in non-stimulatory conditions (culture medium). Normality of the distribution was checked with Shapiro–Wilk test. For comparisons between groups, Mann–Whitney U test was used and differences were considered significant for *p* values < 0.05. Supplementary Figure S4. Production of CXCL13 in human astrocytes of respective donors: D1 (A,B), D2 (C,D), D3 (E,F). Results were acquired from 4 separate experiments performed for 3 donors. Data shown as mean chemokine concentration ± SD. Cells were cultured on 48-well plates for 6 days in proinflammatory conditions (TNF-α/IL-1a/C1q), myricetin stimulation (MYR), chrysin stimulation (CHR), proinflammatory conditions with addition of myricetin (MYR + TNF-α/IL-1a/C1q), proinflammatory conditions with addition of chrysin (CHR + TNF-α/IL-1a/C1q) and in non-stimulatory conditions (culture medium). Normality of the distribution was checked with Shapiro–Wilk test. For comparisons between groups, Mann–Whitney U test was used and differences were considered significant for *p* values < 0.05.
