# Supplementary material for: Dietary Polyphenols Decrease Chemokine Release by Human Primary Astrocytes Responding to Pro-Inflammatory Cytokines
Source: Pharmaceutics. 2023 Sep 7;15(9):2294. doi: 10.3390/pharmaceutics15092294 (PMC10537369; doi:10.3390/pharmaceutics15092294)
Supplement: Supplementary file 1 [file pharmaceutics-15-02294-s001.zip › pharmaceutics-2482266-supplementary.pdf]

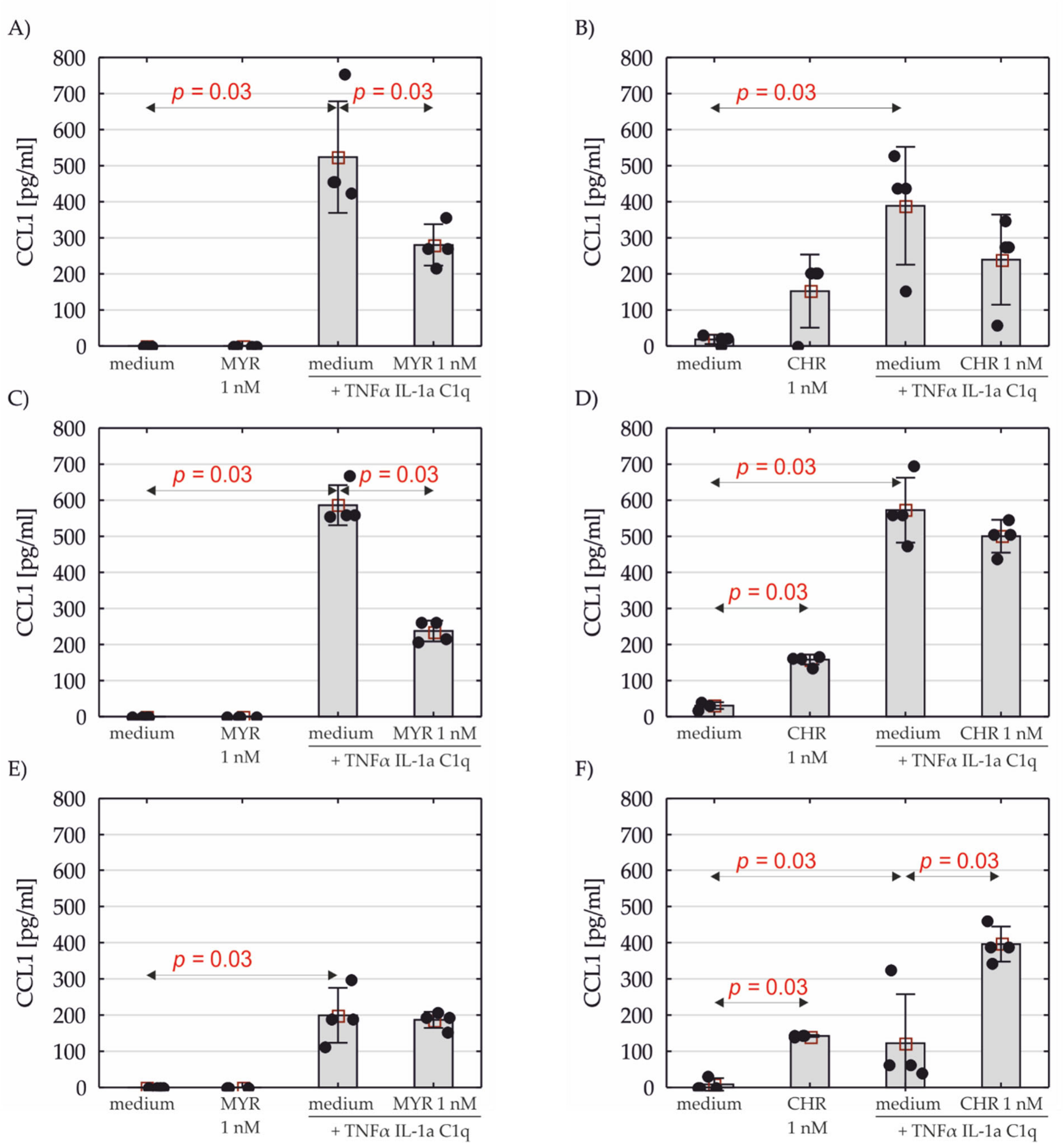

Supplementary Figure S1. Expression of CCL1 in human astrocytes of respective donors: D1 (**A,B**), D2 (**C,D**), D3 (**E,F**). Results were acquired from 4 separate experiments performed for 3 donors. Data shown as mean chemokine concentration ± SD. Cells were cultured on 48-well plates for 6 days in proinflammatory conditions (TNF-α/IL-1a/C1q), myricetin stimulation (MYR), chrysin stimulation (CHR), proinflammatory conditions with addition of myricetin (MYR + TNF-α/IL-1a/C1q), proinflammatory conditions with addition of chrysin (CHR + TNF-α/IL-1a/C1q) and in non-stimulatory conditions (culture medium). Normality of the distribution was checked with Shapiro–Wilk test. For comparisons

between groups, Mann–Whitney U test was used and differences were considered significant for p values < 0.05.

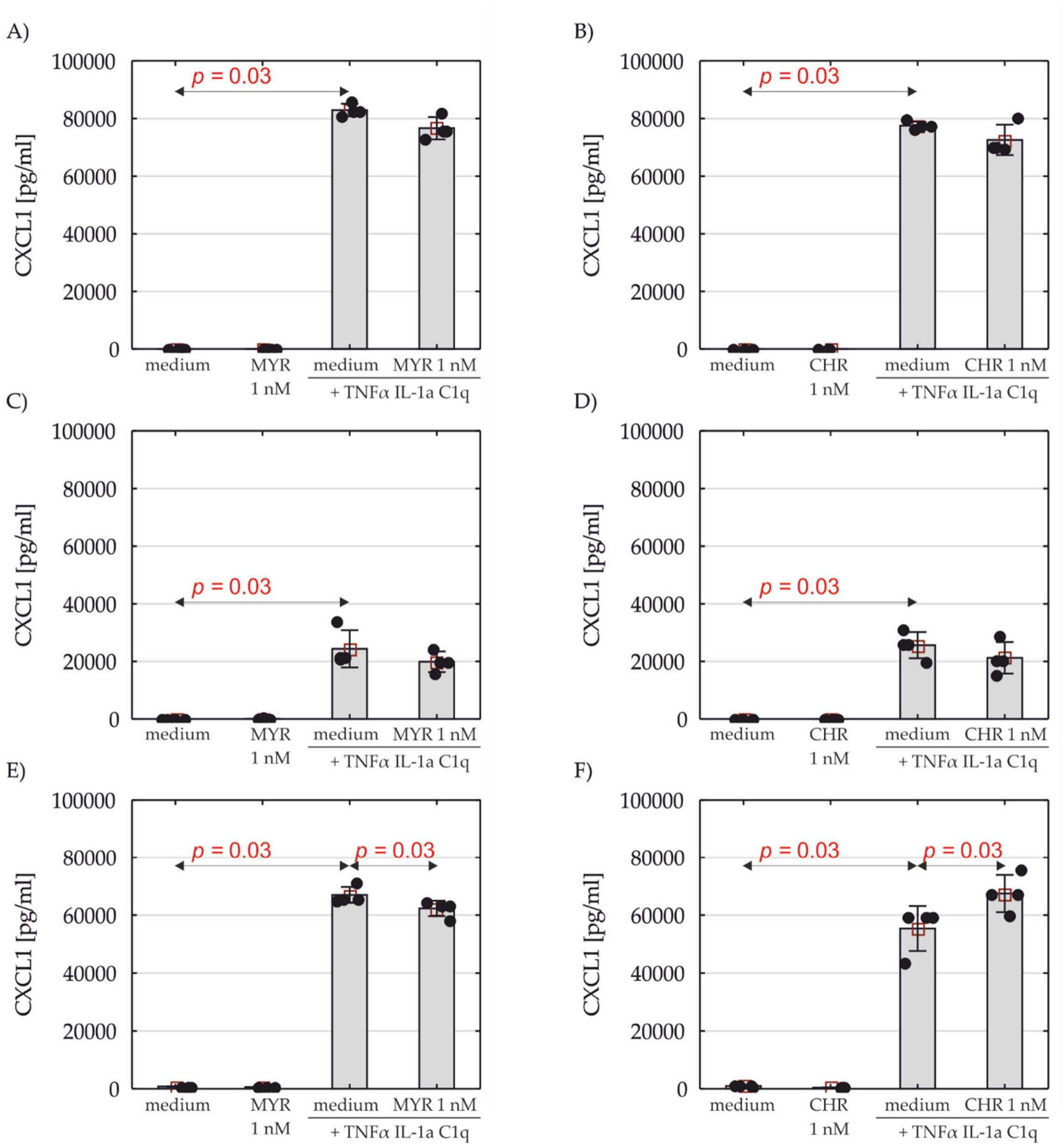

Supplementary Figure S2. Production of CXCL1 in human astrocytes of respective donors: D1 (**A,B**), D2 (**C,D**), D3 (**E,F**). Results were acquired from 4 separate experiments performed for 3 donors. Data shown as mean chemokine concentration ± SD. Cells were cultured on 48-well plates for 6 days in proinflammatory conditions (TNF-α/IL-1a/C1q), myricetin stimulation (MYR), chrysin stimulation (CHR), proinflammatory conditions with addition of myricetin (MYR + TNF-α/IL-1a/C1q), proinflammatory conditions with addition of chrysin (CHR + TNF-α/IL-1a/C1q) and in non-stimulatory conditions (culture medium). Normality of the distribution was checked with Shapiro–Wilk test. For comparisons

between groups, Mann–Whitney U test was used and differences were considered significant for p values < 0.05.

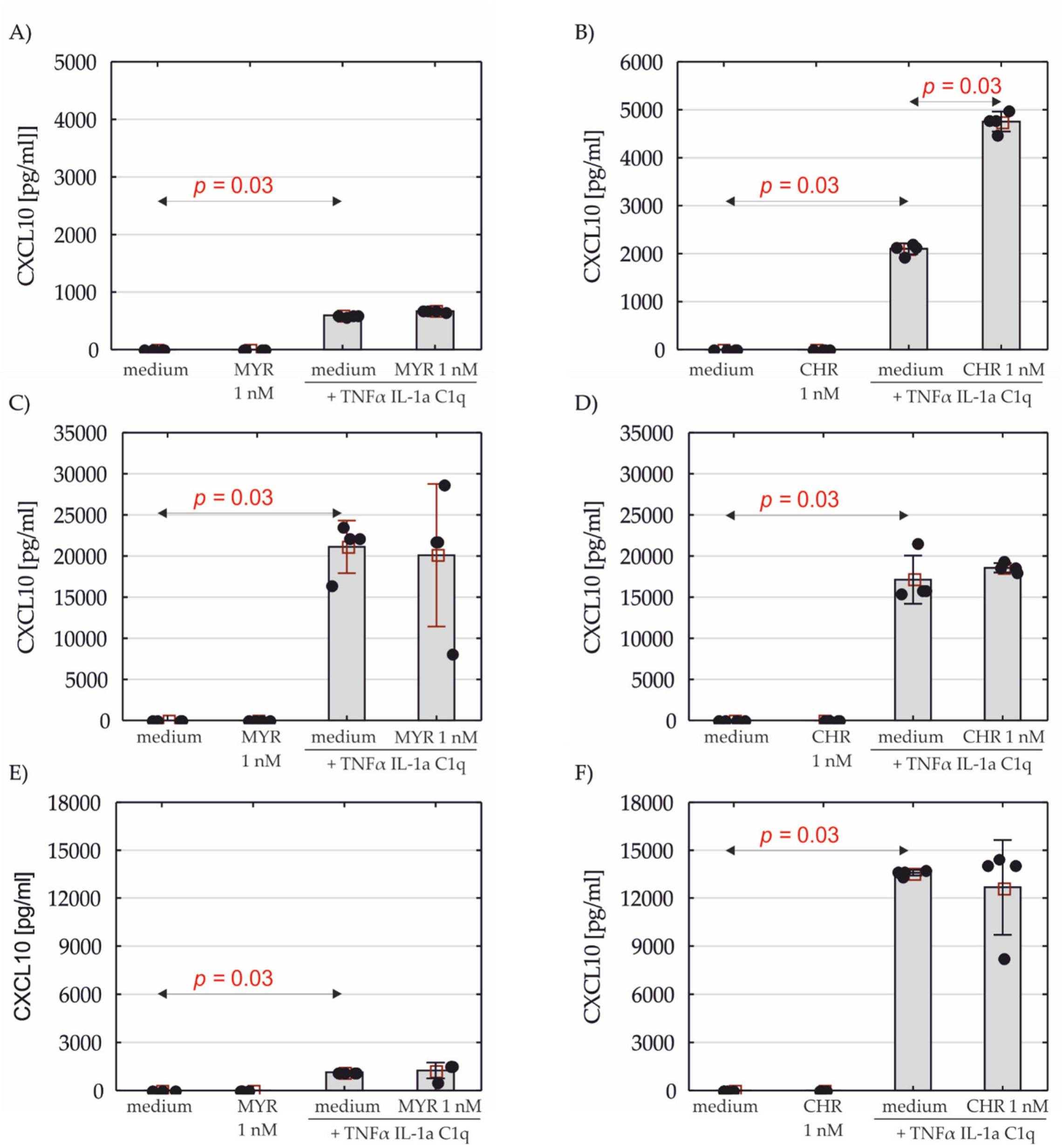

Supplementary Figure S3. Production of CXCL10 in human astrocytes of respective donors: D1 (**A,B**), D2 (**C,D**), D3 (**E,F**). Results were acquired from 4 separate experiments performed for 3 donors. Data shown as mean chemokine concentration ± SD. Cells were cultured on 48-well plates for 6 days in proinflammatory conditions (TNF-α/IL-1a/C1q), myricetin stimulation (MYR), chrysin stimulation (CHR), proinflammatory conditions with addition of myricetin (MYR + TNF-α/IL-1a/C1q), proinflammatory conditions with addition of chrysin (CHR + TNF-α/IL-1a/C1q) and in non-stimulatory conditions (culture medium). Normality of the distribution was checked with Shapiro–Wilk test. For comparisons

between groups, Mann–Whitney U test was used and differences were considered significant for p values < 0.05.

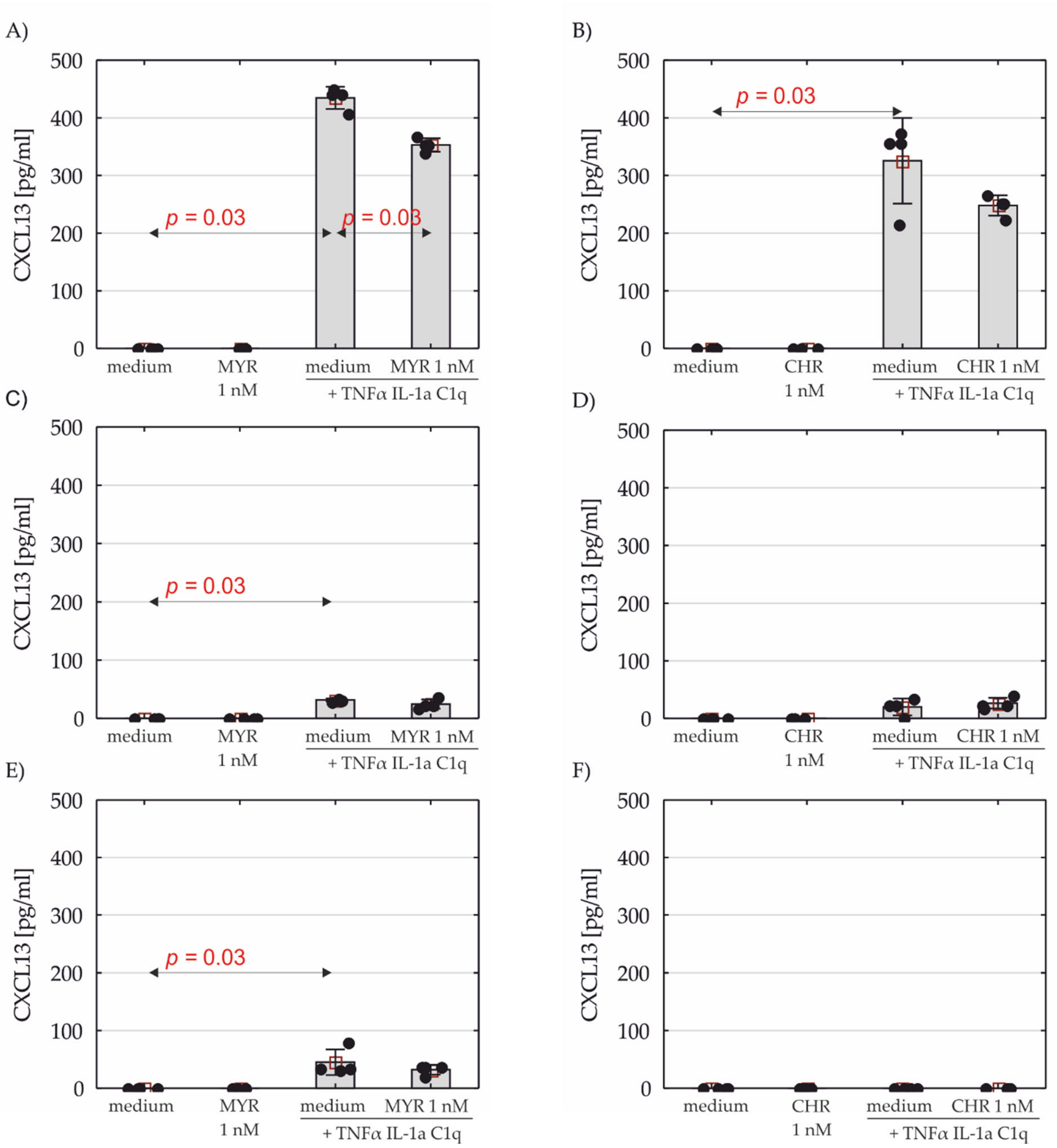

Supplementary Figure S4. Production of CXCL13 in human astrocytes of respective donors: D1 (**A,B**), D2 (**C,D**), D3 (**E,F**). Results were acquired from 4 separate experiments performed for 3 donors. Data shown as mean chemokine concentration ± SD. Cells were cultured on 48-well plates for 6 days in proinflammatory conditions (TNF-α/IL-1a/C1q), myricetin stimulation (MYR), chrysin stimulation (CHR), proinflammatory

conditions with addition of myricetin (MYR + TNF-α/IL-1a/C1q), proinflammatory conditions with addition of chrysin (CHR + TNF-α/IL-1a/C1q) and in non-stimulatory conditions (culture medium). Normality of the distribution was checked with Shapiro–Wilk test. For comparisons between groups, Mann–Whitney U test was used and differences were considered significant for p values < 0.05.
